# Determinism of nonadditive litter mixture effect on decomposition: Role of the moisture content of litters

**DOI:** 10.1002/ece3.7771

**Published:** 2021-06-21

**Authors:** Sébastien Gogo, Fabien Leroy, Renata Zocatelli, Adrien Jacotot, Fatima Laggoun‐Défarge

**Affiliations:** ^1^ Univ. Orléans, CNRS, BRGM, ISTO, UMR 7327 Orléans France

**Keywords:** litter decomposition, litter water content, *Molinia caerulea*, nonadditive effect, peatland, *Sphagnum*

## Abstract

The mechanisms behind the plant litter mixture effect on decomposition are still difficult to disentangle. To tackle this issue, we used a model that specifically addresses the role of the litter moisture content. Our model predicts that when two litters interact in terms of water flow, the difference of evaporation rate between two litters can trigger a nonadditive mixture effect on decomposition. Water flows from the wettest to the driest litter, changing the reaction rates without changing the overall litter water content. The reaction rate of the litter receiving the water increases relatively more than the decrease in the reaction rate of the litter supplying the water, leading to a synergistic effect. Such water flow can keep the microbial biomass of both litter in a water content domain suitable to maintain decomposition activity. When applied to experimental data (*Sphagnum* *rubellum* and *Molinia caerulea* litters), the model is able to assess whether any nonadditive effect originates from water content variation alone or whether other factors have to be taken into account.

## Graphical abstract

The mechanisms behind the plant litter mixture effect on decomposition are still difficult to disentangle. The model proposed in this article predicts that when two litters interact in terms of water flow, the difference of evaporation rate between two litters can trigger a nonadditive mixture effect on decomposition. Water flows from the wettest to the driest litter, changing the reaction rates without changing the overall litter water content.

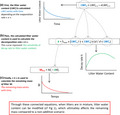

## 1 INTRODUCTION

Litter decomposition is a key process of ecosystem functioning as it affects the amount of soil organic matter (SOM) and the availability of nutrients for hetero‐ and autotrophic organisms (Berg & McClaugherty, 2008). The main factors controlling litter and SOM decomposition are the diversity and biomass of decomposers, climate, and litter quality (Couteaux et al., 1995; Swift et al., 1979). Plant diversity is also a determining factor (Gartner & Cardon, 2004; Hättenschwiler et al., 2005). Litters from the different plant species decompose in situ in mixture, so it is pertinent to study decomposition not only in single‐species context but also in mixture.

The mass loss of litters in mixture can be equal to the average mass loss of each litter decomposing in single‐species situation, weighted by their contribution to the mixture. This is referred to as an additive effect. In other cases called nonadditive, litter mixing induces an increase (synergistic effect) or a decrease (antagonistic effect) of the decomposition rate compared to what can be expected from each litter decomposing in single‐species situation.

The mechanisms behind nonadditive mixture effect can be triggered by the transfer of nutrients (Salamanca et al., 1998; Schimel & Hättenschwiler, 2007), transfer of organic compounds (Schimel et al., 1998), a change in the behavior of soil organisms (Hättenschwiler & Bretscher, 2001), and/or an improvement of micro‐environmental conditions (IMC, Makkonen et al., 2013; Wardle et al., 2003).

For the transfer of nutrients, it is hypothesized that when litters of contrasted quality in terms of nutrient content (e.g., C/N ratio) are decomposing together, the nutrient‐rich litter decomposes faster, leading to an increase of available nutrient. These available nutrients can be transferred to the low‐quality/nutrient‐poor litter, which decomposition would be stimulated by the input of nutrients (Salamanca et al., 1998; Wardle et al., 1997). Such a mechanism may not act alone. For example, Pérez Harguindeguy et al. (2008) showed that enhanced decomposability could be explained by both mean nutrient content of the mixture and its heterogeneity (expressed as nonlabile compound concentrations). Furthermore, other studies did not support this hypothesis by showing either no synergistic effect of mixture in litter with contrasted C/N ratio (Klemmedson, 1992) or synergistic effect of mixture in litter with similar C/N ratio (Hoorens et al., 2010).

Some chemical compounds are known to inhibit microbial decomposition (e.g., condensed tannins in *Populus basalmifera* litter, Schimel et al., 1998). Thus, when litters containing such allelopathic compounds decompose in mixture with another litter, these compounds can flow from one litter to another and the decomposition of the latter could be reduced (antagonistic effect). Such an effect could interact with nutrients (Zhang et al., 2014; Zhou et al., 2020). However, such study is still scarce.

Improvement of micro‐environmental conditions hypothesis states that, in mixture, one litter improves the physical conditions for micro‐organisms, which induces an increase of the decomposition of the other litter. Maintaining optimal water content is crucial as too much water can prevent oxygen diffusion (inducing a decrease in microbial activity) and not enough water can stop many metabolic pathways. This could be achieved in litter with high water holding capacity (the maximum amount of water per unit of dry mass a litter can hold). Among the physical factors, improvement of water availability in litter with contrasted water holding capacities was found to be promote synergistic effect in limiting moisture conditions (Makkonen et al., 2013). However, they showed that when moisture is no longer limiting, antagonistic effects occur.

Overall, not a single mechanism explains the observed nonadditive mixture effect on litter decomposition. They combine and interact to produce the in situ and laboratory observations. It is probably the interplay of these mechanisms that could generate apparent contradictory results. Thus, to untangle the mechanisms behind nonadditive effect and to try to highlight how they can interact, a more generic approach through modeling should be initiated. The first mechanism that could be modeled, by taking into account the water properties of litters (water content, water holding capacity), is the IMC hypothesis.

Improvement of micro‐environmental conditions related to water availability may be the mechanism most profoundly impacted by climate change, through projected changes in drought duration and the amount of precipitation (IPCC, 2013). As physical properties affecting the water content differ from one litter to another (Makkonen et al., 2013), litters may not behave in the same way toward changes in soil moisture regime. Thus, the role of dynamics of the litter water content in explaining the litter mixture effect should be examined in detail.

Some mosses such as *Sphagnum* mosses and feather mosses are known to be efficient in retaining water with a potential effect on decomposition of the litter in mixture (Hoorens et al., 2002, 2010; Wardle et al., 2003). Gogo et al. (2016) previously demonstrated that a nonadditive synergistic litter mixture effect on decomposition occurs in situ when *Sphagnum* *rubellum* litter decomposes with *Molinia caerulea* litter. *Molinia caerulea* is an invasive species that could colonize vast areas of north‐west European peatlands and heathlands (Chambers et al., 1999). They mix with Sphagnum and tend to overcompete the moss in many cases. *Molinia caerulea* water holding capacity is low compared to *Sphagnum* species. Such difference can influence decay rates in mixture. In a laboratory experiment, Gogo et al. (2016) tested the hypothesis that *S*. *rubellum* litter could promote decomposition by maintaining higher water content in the litter mixture compared to the litter water that could be the expected calculated from the litter incubated alone. As *M*. *caerulea* litter has a low water holding capacity compared to *Sphagnum* spp., even a low drop of litter water content would place the litter in a situation where lack of water availability would limit microbial activity. When in mixture, *Sphagnum* spp. should keep *M*. *caerulea* litter moist enough to support decay. Although Gogo et al. (2016) showed a nonadditive effect of mixing *S*. *rubellum* and *M*. *caerulea* on litter decomposition in the laboratory, they did not find any significant difference between expected and observed whole mixture water content. This lack of nonadditive effect of mixture on the whole litter water content (LWC) ruled out the role of the total water content of the mixture in the nonadditive effect on decomposition (Gogo et al., 2016). However, if water can flow from the wettest litter to the driest one (as in the nutrient‐based mechanisms, Schimel & Hättenschwiler, 2007), then the wettest litter becomes drier and the driest litter becomes wetter, without changing the whole water content of the mixture. Microbial activity involved in litter decomposition is sensitive to LWC (Bunnell et al., 1977; Moyano et al., 2013). Then, the modification of LWC of the different litters in mixture caused by water transfer (situation “with interaction” between litters) could increase or decrease the decomposition rate of litters in mixture compared to a situation where water flow do not occur (situation with “no interaction” between litters). With this flow of water, each microbial biomass could be maintained in a litter moisture domain, where the microbial activity can maintain and avoid inhibition caused by a lack of water. Thus, depending on the physical and biological characteristics of each decomposing litter in a mixture, a nonadditive effect could be expected with exchange of water between litters. These characteristics are (a) litter organic matter (OM) decay sensitivity to water content, (b) initial water content, and (c) litter evaporation rate.

The first objective of this study was to test theoretically whether different dynamics of the water content of decomposing litters in a mixture can produce nonadditive litter mixture effect. Water availability is compulsory for biological activity, but water saturation dramatically reduces oxygen diffusion. Thus, we hypothesized that the double inhibitory effect on microbial activity at each end of the litter water content range, which determines the shape of the sensitivity of decay rate to water availability (inhibiting/limiting at both extremities of the litter water content and optimal with medium values), could be at the origin of nonadditive effect on decomposition. The second objective was to compare modeled data to experimental observations. The second goal comprised two steps: (a) calibration of the model with data from two litters decomposing alone (*S*. *rubellum* and *M*. *caerulea*) and (b) calculation of the remaining mass of the litters in mixture, by using the parameters obtained with the calibration and comparison of the modeled data with the observations (validation). To achieve this goal, published data from Gogo et al. (2016), documenting the nonadditive mixture effect on litter decomposition of these two peatland species, were used.

## 2 MATERIALS AND METHODS

### 2.1 Description of the previous experimental setup and of the numerical approach

The laboratory experiment in Gogo et al. (2016) that incite the construction of the model is briefly presented to better understand the modeling approach that follows. In the laboratory, litters of *Sphagnum* *rubellum* (*S. rub*.) and *Molinia caerulea* (*M*. *cae*.) were incubated in glassware in three different configuration: *S. rub*. alone, *M*. *cae*. alone, and *S. rub*. and *M*. *cae*. in mixture (50% each). A total of 126 samples (42 of each type) were prepared and incubated in the same conditions. Groups of one sample from each type (*S. rub*. + *M*. *cae*. + mix) were composed. A kinetic study was undertaken with the random retrieval of six groups of samples at seven dates (18 samples per date). Whole sample water content, remaining mass, water extractable organic matter, and the CO_2_ production were measured at each date. *S. rub*. and *M*. *cae*. were chosen because a litter bag experiment in the field showed that the mixture of these two litters promoted a synergistic effect. *S. rub*. has a higher water content and a lower decomposability compared to *M*. *cae*.

As the synergistic effect was not triggered by a higher LWC in the mixture compared to what can be expected, it can be hypothesized that water can be exchanged between litters. Litter decomposition is affected by water content, but not in a linear way (Bunnell et al., 1977). Decomposition is at its lowest at the low and high water content and reach a plateau at medium water content. Thus, depending on the water content of each litter in the mixture, water can flow from the wettest litter without affecting much its decomposability (plateau situation), while it can dramatically affect the driest litter decomposition. This will move the driest litter from a situation where decomposition is limited by water content to a situation where water is no longer limiting. This can happen without changing the whole LWC of the mixture. In the Gogo et al. (2016) experiment, *S. rub*. is the litter that has a high LWC and can lose water without much modification of its decomposition rate, while the *M*. *cae*. is the dry litter that decomposition rate can benefit from an input of water.

To test this hypothesis that can be applied to many other litters than *S. rub*. and *M*. *cae*., a numerical approach was chosen. The numerical experiments allow testing the theoretical plausibility of a nonadditive effect on litter decomposition triggered by a flow of water from one litter to another. The numerical experiments consisted in making a relation, or not, between two decomposing litters in mixture by allowing the wettest litter (WET‐LIT, like *S. rub*. in the laboratory experiment) to moisten the driest one (DRY‐LIT, like *M*. *cae*.) and thus change the decomposition rate through a change in the LWC at the individual litter level.

**FIGURE 1:**
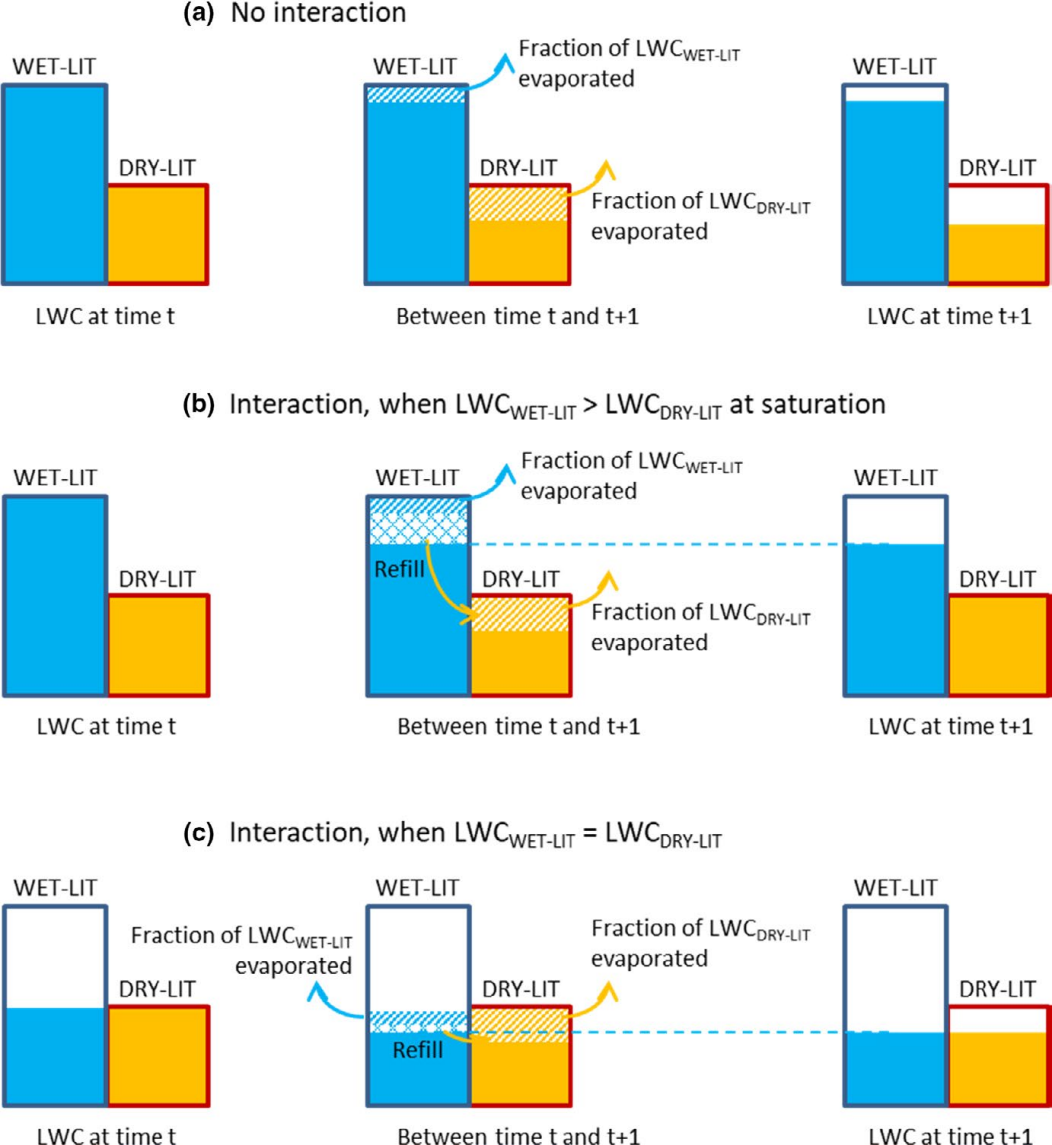
Schematic diagram showing the “no interaction” (a, no water flow from one litter to another) and “with interaction” (b and c, with water flow) situations between two litters with different hydric properties (maximum litter water content or LWC_max_, evaporation rate). Boxes represent the maximum water content for each litter (LWC_max_). WET‐LIT has a high LWC and a low evaporation rate. DRY‐LIT has a low LWC and a high evaporation rate. The flows of water are shown when LWC_WET‐LIT_ is greater than LWC_DRY‐LIT_ (b) and when it is equal (c)

Two types of mixtures were simulated, differing in terms of water transfer between litters. In the mixture with "no interaction," the two litters decomposed without any transfer of water from the wettest litter WET‐LIT to the driest litter DRY‐LIT (Figure 1a). The “no interaction” corresponds to the expected data in the laboratory experiment, calculated from the results obtained with the litters decomposing alone. In the mixture with "interaction," it was assumed that water flowed from WET‐LIT to the DRY‐LIT, after the water in each litter evaporates at its specific evaporation rate (Figure 1b,c). As long as WET‐LIT remained the wettest, it "refilled" DRY‐LIT till its LWC_max_ (Figure 1b). After the two litters reached the same LWC, the average LWC between the two litters, after specific evaporation, was attributed to both litters (Figure 1c).

**FIGURE 2:**
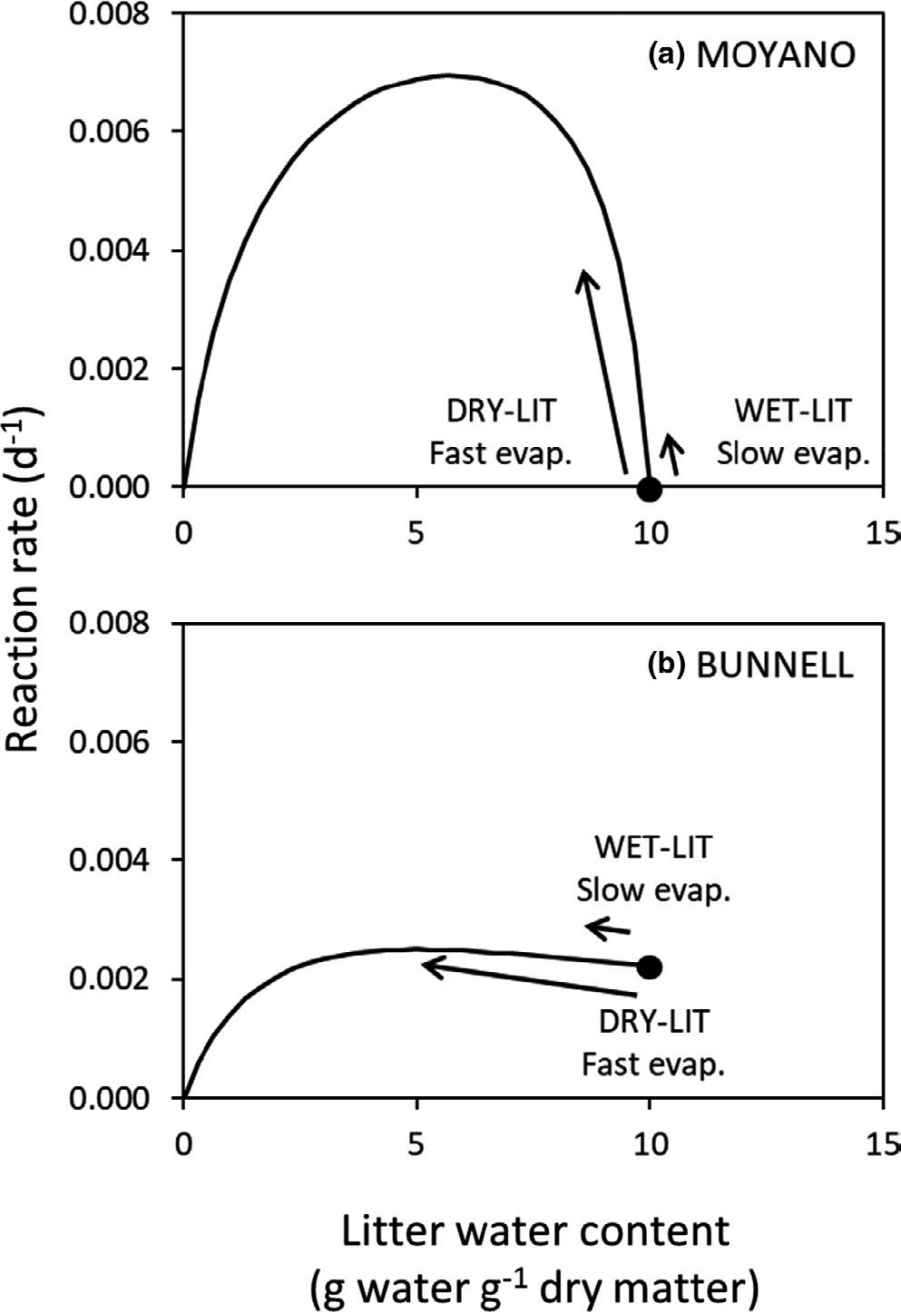
Relationship between litter water content (LWC, g water g^−1^ dry matter) and reaction rate set in the first numerical experiment “Model comparison,” corresponding to (a) the Moyano et al. (2013) model and (b) the Bunnell et al. (1977) model. Water from DRY‐LIT evaporates faster than water in WET‐LIT. The black dot represents the initial LWC of the simulations, which corresponds to the LWC_max_ of each litter, and the length of the arrows is proportional to the evaporation rate

In the first numerical experiment named “Model Comparison,” two representations of the relationship between decomposition rate and LWC were compared: (a) the Moyano et al. (2013) approach and (b) the Bunnell et al. (1977) approach (Figure 2, see following section for details). The same sensitivity to LWC and the same initial water content (LWC_max_) were used for both litters. Only the evaporation rate differed between WET‐LIT (slow evaporation) and DRY‐LIT (fast evaporation).

**FIGURE 3:**
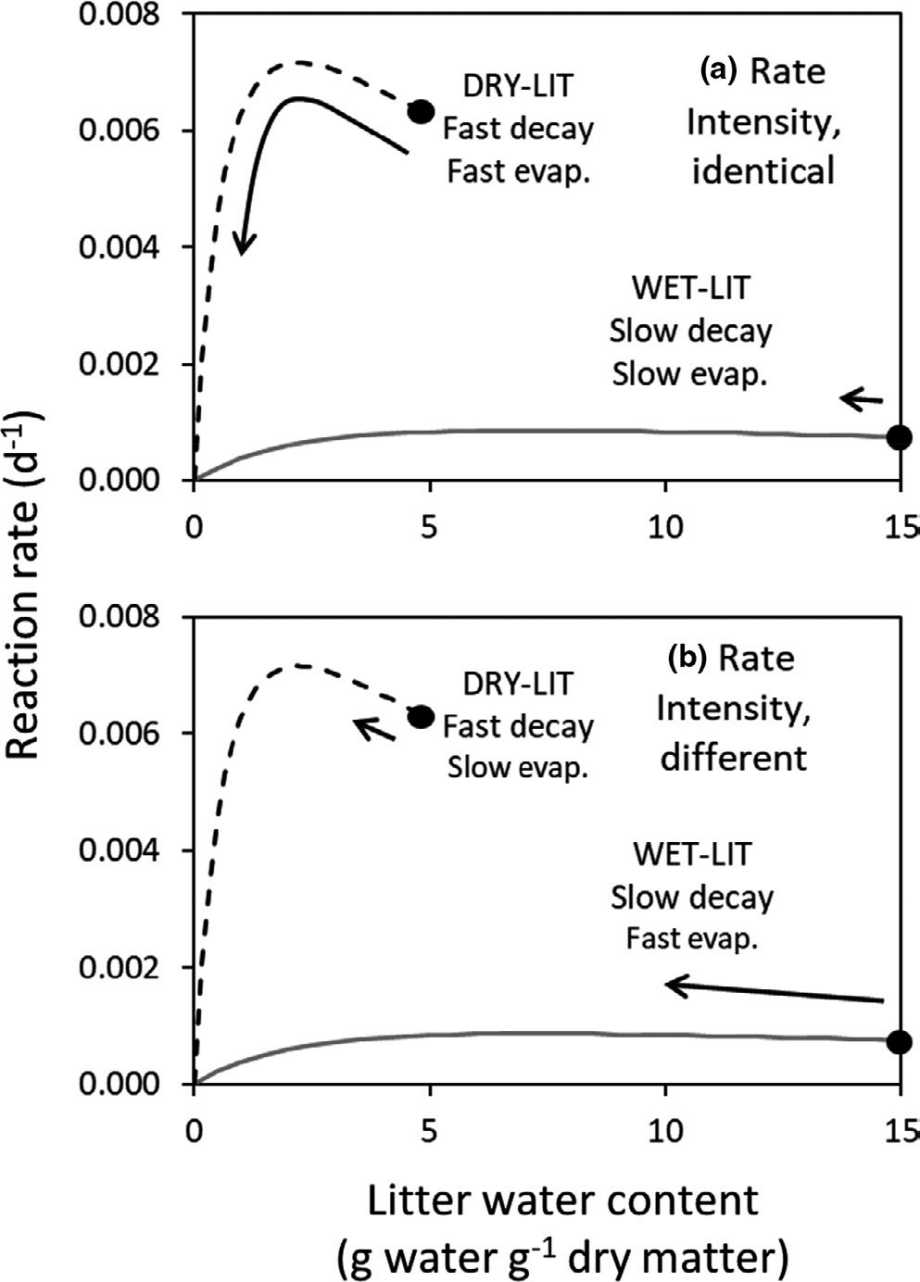
Relationship between litter water content (LWC, g water g^−1^ dry matter) and reaction rate set in the second numerical experiment “Rate combinations” using the Bunnell et al. (1977) model with different combinations of decay and evaporation rates of the litters in the mixture: (a) Identical rate intensity: WET‐LIT decomposes (black dot low) and evaporates (small arrow) slowly + DRY‐LIT decomposes (black dot high) and evaporates (long arrow) rapidly. (b) Different rate intensity: WET‐LIT decomposes slowly (black dot low) and evaporates rapidly (long arrow) + DRY‐LIT decomposes rapidly (black dot high) and evaporates slowly (long arrow)

In the second numerical experiment named “Rate Combinations,” the effect of combining different rate of decay and evaporation on the nonadditive effect was tested (Figure 3). WET‐LIT and DRY‐LIT had a decomposition rate with a different sensitivity to LWC and a different initial water content (LWC_max_) that did not change between the different simulations. Two simulations were performed with only a modification of the evaporation rate. In the first case, DRY‐LIT evaporated faster than WET‐LIT (Figure 3a), and in the second case, DRY‐LIT evaporated slower than litter 2 (Figure 3b). Simulations of the Rate Combinations numerical experiment were performed with one type of model. As the validation was made with a laboratory litter decomposition experiment, the model that best represents the LWC sensitivity in this kind of material should be used. The exchange surface with air in litter is much higher than in soil and, thus, a significant amount of oxygen can be available. Thus, the Bunnell et al. (1977) approach was preferred, because it allows the microbial biomass to be active at saturation.

### 2.2 Model description

**FIGURE 4:**
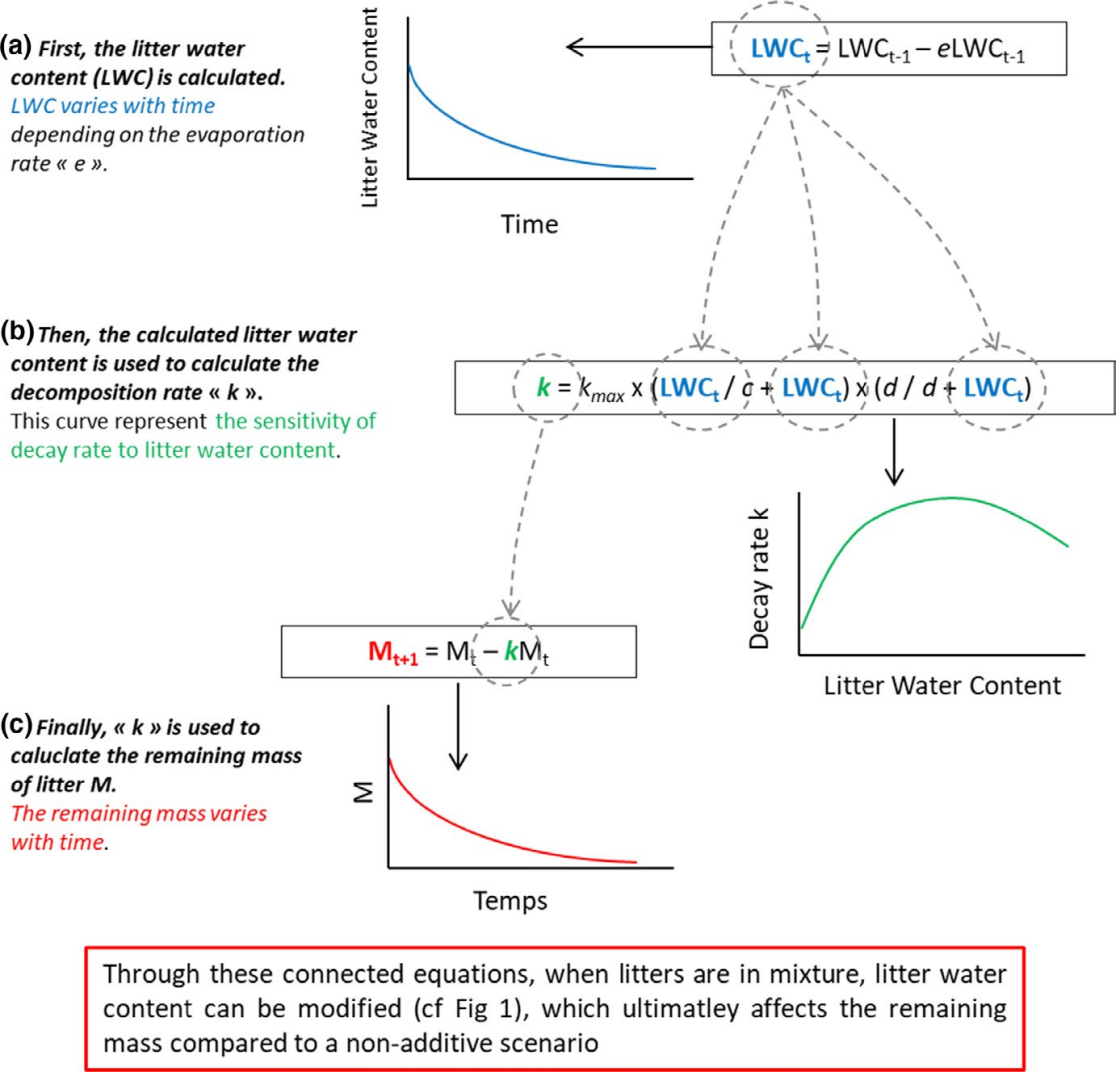
Schematic diagram showing the model relating litter water content to evaporation rate, the reaction rate to the litter water content, and the remaining mass to the change of reaction rate with time, with LWC: litter water content, *k*: decomposition rate, *M*: remaining mass, *c*, *d*, *e*: empirical parameters, and *k*_max_: maximum decomposition rate

The time step of all the discrete equations is the day. The relationship between the different equations is illustrated in Figure 4. Equation 1 was used to model the decomposition of OM with time (discretization of the negative exponential decay model), with parameter *k* as the decomposition rate.

(1) $$M_{t+1}=M_{t}‐kM_{t}$$

*M_t_*_+1_ represents the remaining mass of litter at time “*t* + 1 day,” *M_t_* is the mass of litter at time “*t*” and *k* is the decomposition rate. Concretely, *k* is the fraction of litter mass present at time *t* (*M_t_*) that was lost between time *t* and *t* + 1 day.

To account for the effect of the properties of litter related to water content (maximum water content, varying water content with time) on the decomposition rate, the relationship between *k* and litter water content (LWC) as well as the evaporation rate was modeled. In a first step, two models of OM decomposition, which incorporate sensitivity to water content in different ways, were compared. Both models used two Michaelis–Menten equations to account for the simultaneous dependence of the biological processes on water and oxygen. The more water available, the less oxygen, and vice versa. However, the two models differ by their representation of the relationship to oxygen. The first one was derived from Moyano et al. (2013, Equation 2), which models soil OM and not specifically litter. This model assumes no biological activity at water saturation and uses oxygen as a substrate. Conversely, the Bunnell et al. (1977) model allows biological activity at water saturation. It derives from a Michaelis–Menten equation and LWC a coefficient, which describes how the gas exchange is facilitated. This coefficient is maximal at low LWC, but is not necessarily equal to zero at saturation.

(2) $$k_{\text{Moyano}}=k_{\text{max}}\times \frac{\text{LWC}}{a+\text{LWC}}\times \frac{O_{a}}{b+O_{a}}$$

(3) $$k_{\text{Bunnell}}=k_{\text{max}}\times \frac{\text{LWC}}{c+\text{LWC}}\times \frac{d}{d+\text{LWC}}$$

with *O_a_* represented as follows (Equation 4):

(4) $$O_{a}=\frac{{\text{LWC}}_{\text{max}}‐\text{LWC}}{{\text{LWC}}_{\text{max}}}$$

LWC_max_ is the maximum water content for a specific litter (water saturation) and *a*, *b*, *c*, and *d* are the Michaelis–Menten constants (the LWC when the reaction rates are equal to half of the *k*_max_). *O_a_* is a coefficient calculated to allow the decomposition rate to be equal to zero at maximum LWC (when LWC = LWC_max_, *O_a_* = 0 and thus *k* = 0). Finally, to represent LWC with time, we used a discrete negative exponential decay model, with parameter *e* as the rate at which water is lost (the fraction of water present at time *t* − 1 lost between time *t* − 1 and *t*, Equation 5).

(5) $${\text{LWC}}_{t}={\text{LWC}}_{t‐1}‐{\text{eLWC}}_{t‐1}$$

At the start of each numerical experiment, the LWC of each litter was set at its maximum (LWC_max_). At the beginning of each time step, LWC was allowed to change depending on the evaporation rate *e* (Equation5; Figure 4a), which was allowed to differ between species (Figure 1). The calculated LWC was then injected into Equation 3 (Figure 4b) and 4 to calculate *k*_Bunnell_ and *O_a_*, respectively, and then *O_a_* was used to calculate *k*_Moyano_ (Equation2). The *a*, *b*, *c*, and *d* parameters of the Equations 3 and 4 differed, or not, between the two litters. Each calculated *k* was finally injected into Equation 1 to obtain the remaining mass at the end of each time step for each litter and for each model type (Figure 4c). The parameters used in the “Model Comparison” and in “Rate Combinations” numerical experiments are listed in Table 1.

**TABLE 1:** Values of the parameters used in the Model Comparison and Rate Combinations experiments (LWC_max_ = maximum litter water content in g of water g^−1^ of dry mass, *k*_max_ and e in d^−1^, *a*, *b*, *c,* and *d* in g of water g^−1^ of dry mass, and n.a. = not applicable)

Parameter	Model Comparison				Rate Combinations			
	Moyano model		Bunnell model		Identical within litter		Different within litter	
	WET‐LIT	DRY‐LIT	WET‐LIT	DRY‐LIT	WET‐LIT	DRY‐LIT	WET‐LIT	DRY‐LIT
LWC_max_	10	10	10	10	15	5	15	5
*k*_max_	0.01	0.01	0.01	0.01	0.005 (*low*)	0.015 (**HIGH**)	0.005 (*low*)	0.015 (**HIGH**)
*a*	5	5	n. a.	n. a.	n. a.	n. a.	n. a.	n. a.
*b*	0.5	0.5	n. a.	n. a.	n. a.	n. a.	n. a.	n. a.
*c*	n. a.	n. a.	5	5	10	1	10	1
*d*	n. a.	n. a.	5	5	5	5	5	5
*e*	0.01	0.1	0.01	0.1	0.01 (*low*)	0.1 (**HIGH**)	0.1 (**HIGH**)	0.01 (*low*)

Note

HIGH and low in brackets refer to the relative intensity of the different rates.

### 2.3 Calibration

To assess whether the theoretical results obtained in the model can be in accordance with experimental results, the model was fitted to laboratory data obtained by Gogo et al. (2016). In this experiment, the mixture of the wet, slow decomposing litter of *S*. *rubellum* (equivalent to WET‐LIT) with the dry, fast decomposing litter of *M*. *caerulea* (equivalent to DRY‐LIT) generated a synergistic effect on decomposition.

First, the equation of litter water content loss with time (Equation 5) was fitted to the data to obtain the *e* parameter for each litter alone (Table 2; Supplementary Material S1). The initial litter water contents were set based on the first water content measured after 2 days of incubation (measured value rounded up to the integer, e.g., 21.56 gave 22).

**TABLE 2:** Values of the parameter set (LWC_max_ = maximum litter water content in g of water g^−1^ of dry mass) and obtained (*k*_max_ and *e* in d^−1^ and *c* and *d* in g of water g^−1^ of dry mass) by fitting the model to the experimental data of the decomposition of *Sphagnum* *rubellum* and *Molinia caerulea* alone

	*Sphagnum rubellum*	*Molinia caerulea*
LWC_max_	22	3
*k*max	0.0055	0.0060
*c*	22	3
*d*	22	3
*e*	0.010	0.061

Then, the equation of the remaining mass after decomposition (Equation 1) was fitted to the measured remaining mass of each litter decomposing alone (Supplementary Material S2). To do so, the decay rate *k* in Equation 1 was allowed to vary according to Equation 3 (Bunnell et al., 1977 model). To determine *k*, the litter water content (LWC) modeled in the first phase was used. It is the *k*_max_, *c*, and *d* parameters that were tuned to adjust the modeled remaining mass to those measured.

The equations were adjusted to the observed data by minimizing the square difference between modeled and observed data (least square difference technique) with the solver function in Excel. To avoid issues of nonconvergence or unrealistic values, *k*_max_, *c*, and *d* were set superior to zero and the two latter parameters were set to be inferior to LWC_max_ value (22). All the fitted parameters are listed in Table 2.

### 2.4 Validation

To assess whether or not water flow between litters can explain observed nonadditive effect of litter mixture on decomposition, one has to compare results in mixture predicted by the model to real data. To obtain expected mixture results from the model that will be compared to observed mixture results, we used the parameters obtained for each litter in the calibration phase (Table 2). First, to have the “no interaction” data, where water does not flow between litters, we calculated the mass loss of litters alone and summed 50% of these remaining masses from each litter to compose the modeled “no interaction” mixture results. To have the modeled “interaction” mixture results, at each time step water was allowed to flow from *S*. *rubellum* litter (the wet one) to *M*. *caerulea* litter (the dry one), with the rules detailed in Figure 1.

In the last step of the calibration phase, the predicted remaining masses were compared to the measurements made in the laboratory (Gogo et al., 2016). To assess the goodness of fit of the validation, the adjusted *R* ^2^ and the normalized random mean square error (NRMSE) were calculated. The difference between 1) the no interaction and interaction scenarios and between 2) the measured expected remaining mass (calculated from the litter decomposing in a single‐species situation) and the observed remaining mass was calculated.

## 3 RESULTS

### 3.1 Numerical experiments

**FIGURE 5:**
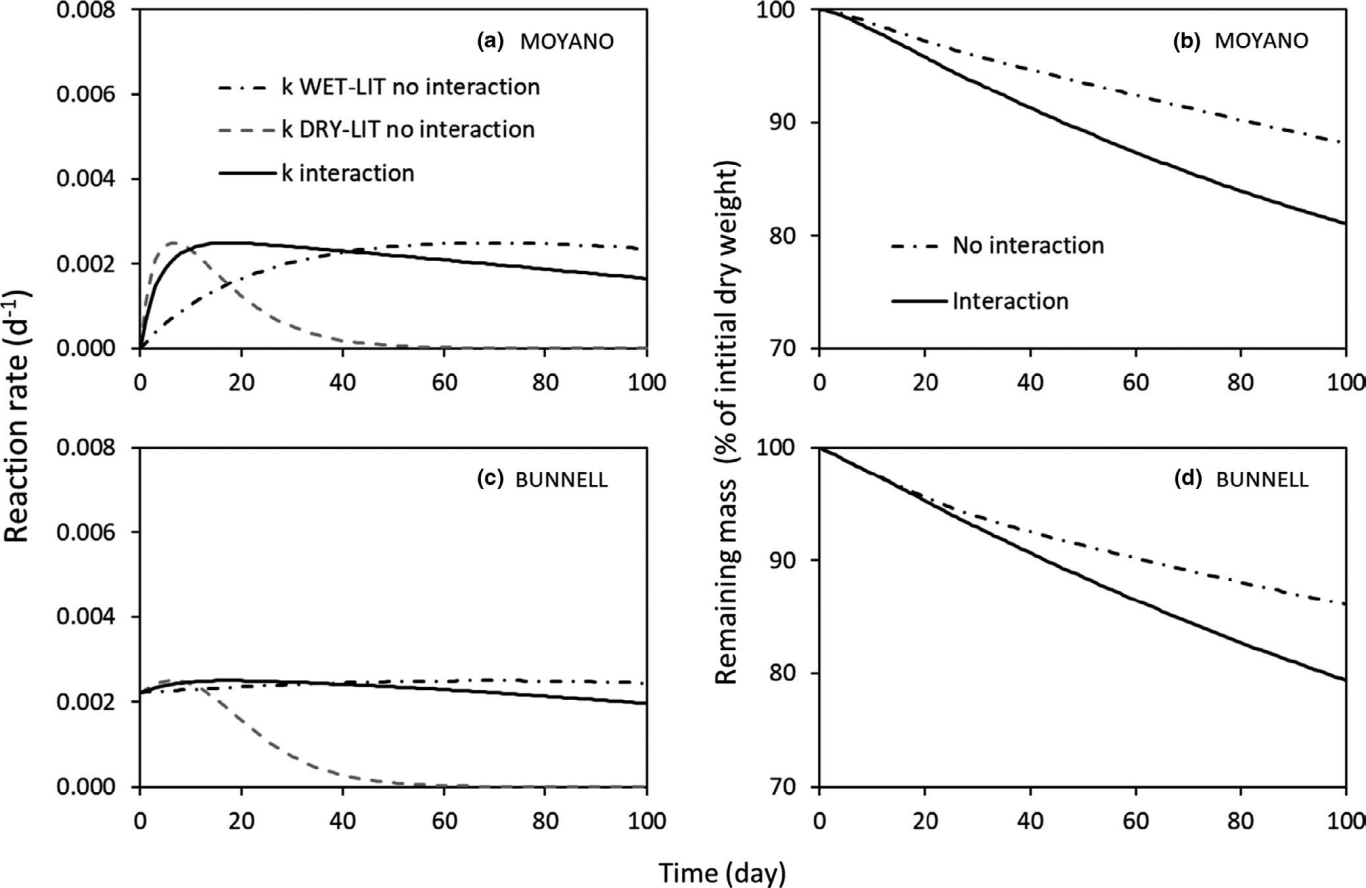
Reaction rates (*k*, d^−1^) and remaining mass (% of initial dry weight) from the Model Comparison experiment. To represent the relationship between the reaction rate and the LWC, equations from Moyano et al. (2013, Equations 2 and 4, a and c) and from Bunnell et al. (1977, Equation 3, b and d) were used. The parameters *k*_WET‐LIT_ and *k*_DRY‐LIT_ are the decomposition rate of the wettest litter and the driest litter, respectively, in the “no interaction” scenario, and *k* is the overall decomposition rate when water is allowed to flow from WET‐LIT to DRY‐LIT

In the Model Comparison experiment (Figure 5), WET‐LIT evaporated at a slower rate than DRY‐LIT. The sensitivity of the decomposition rate to the LWC was the same for the two litters in each simulation, and two different models representing this sensitivity were used (Figure 2). In this context, allowing the water to flow from the wettest litter to the driest one maintained wet conditions in both litters. After 40 days, the decomposition rate of the wettest litter (*k*_WET‐LIT_) in the “no interaction” scenario was higher than with interaction with both models (Figure 5a,c). However, the decrease of *k*_WET‐LIT_ caused by the flow of water from WET‐LIT to DRY‐LIT was small compared to the increase of the decomposition rate observed for the driest litter *k*_DRY‐LIT_ (Figure 5a,c). The overall increase in the decomposition rate in the mixture with interaction increases the mass loss compared to the “without interaction” scenario (Figure 5b,d). Thus, allowing the water to flow from the wettest to the driest litter led to a synergistic effect on litter decomposition, compared to the mixture with no interaction (additive) with the two models tested (Figure 5b,d).

**FIGURE 6:**
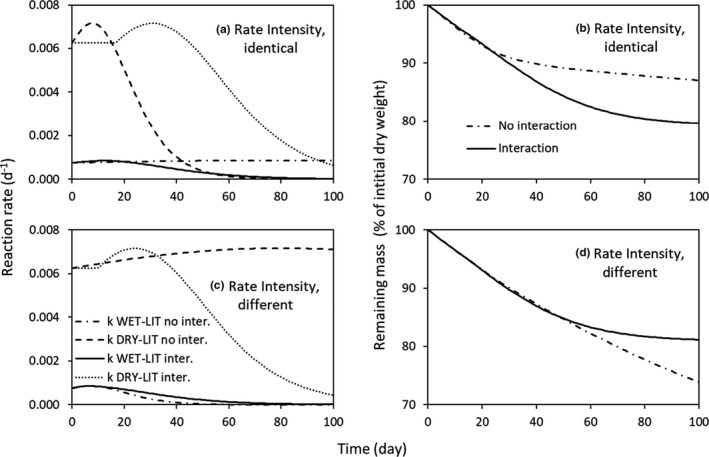
Reaction rates (*k*, d^−1^) and remaining mass (% of initial dry weight) from experiment 2 simulation 1 (a, b, Ex2 Sim1), simulation 2 (Fig. c, d, Ex2 Sim2), and simulation 3 (e, f, Ex2 Sim3). To represent the relationship between the reaction rate and the LWC, Equation 3 from Bunnell et al. (1977) was used in all simulations

In the Rate Combinations numerical experiment, WET‐LIT had a higher LWC_max_ and a lower *k*_max_ than DRY‐LIT (Figure 6; Table 1). In the first simulation where rate intensities were identical, WET‐LIT evaporated slowly, while DRY‐LIT lost water at a faster rate (Table 1). When interaction was allowed, the water rapidly lost by DRY‐LIT was replaced by water from WET‐LIT. As a result, early in the time course of the experiment, the LWC of DRY‐LIT was kept in a water content domain where the reaction rates remained high compared to the situation with “no interaction” (Figure 6a). This increase in reaction in DRY‐LIT largely compensated the decrease of the reaction rate in WET‐LIT caused by the more rapid loss of water in the interaction scenario (flow of water from litter 1 to litter 2) compared to the "no interaction" situation (no water exchange, Figure 6a). Consequently, the remaining mass in the mixture with interaction was lower than in the mixture with no interaction, highlighting a synergistic effect of mixing the two litters (Figure 6b). In the second simulation where rate intensities were different, WET‐LIT evaporated faster than DRY‐LIT (Table 1). In the mixture with interaction, the small water stock contained within DRY‐LIT was quickly consumed to fill the rapidly evaporating WET‐LIT. Consequently, the reaction rate of DRY‐LIT decreased rapidly in the mixture with interaction, while the reaction rate remained high throughout the simulation when water was not allowed to flow from one litter to another (Figure 6c). The decrease in the decomposition rate of DRY‐LIT was not compensated by the relative increase of WET‐LIT decomposition rate in the mixture with interaction (Figure 6c). Thus, the remaining mass in the mixture was higher than in the mixture without interaction, highlighting an antagonistic effect of mixing the two litters (Figure 6d).

### 3.2 Calibration

Fitting the equations (version using the Bunnell et al., 1977 equation) to the results of single litter (no mixture) obtained by Gogo et al. (2016) showed that water in *M*. *caerulea* litter evaporated faster than water in *S*. *rubellum* litter (Table 2; Supplementary Material S1). Furthermore, *k*_max_ of *M*. *caerulea* was higher than that of *S*. *rubellum* (Table 2). However, as *S*. *rubellum* was wetter and evaporated more slowly than *M*. *caerulea* litter, the litter decomposition rate of the gramineae decreased faster than the one of the bryophyte litter. Thus, the remaining mass of *M*. *caerulea* was higher than that of *S*. *rubellum* (Supplementary Material S2).

### 3.3 Validation

**FIGURE 7:**
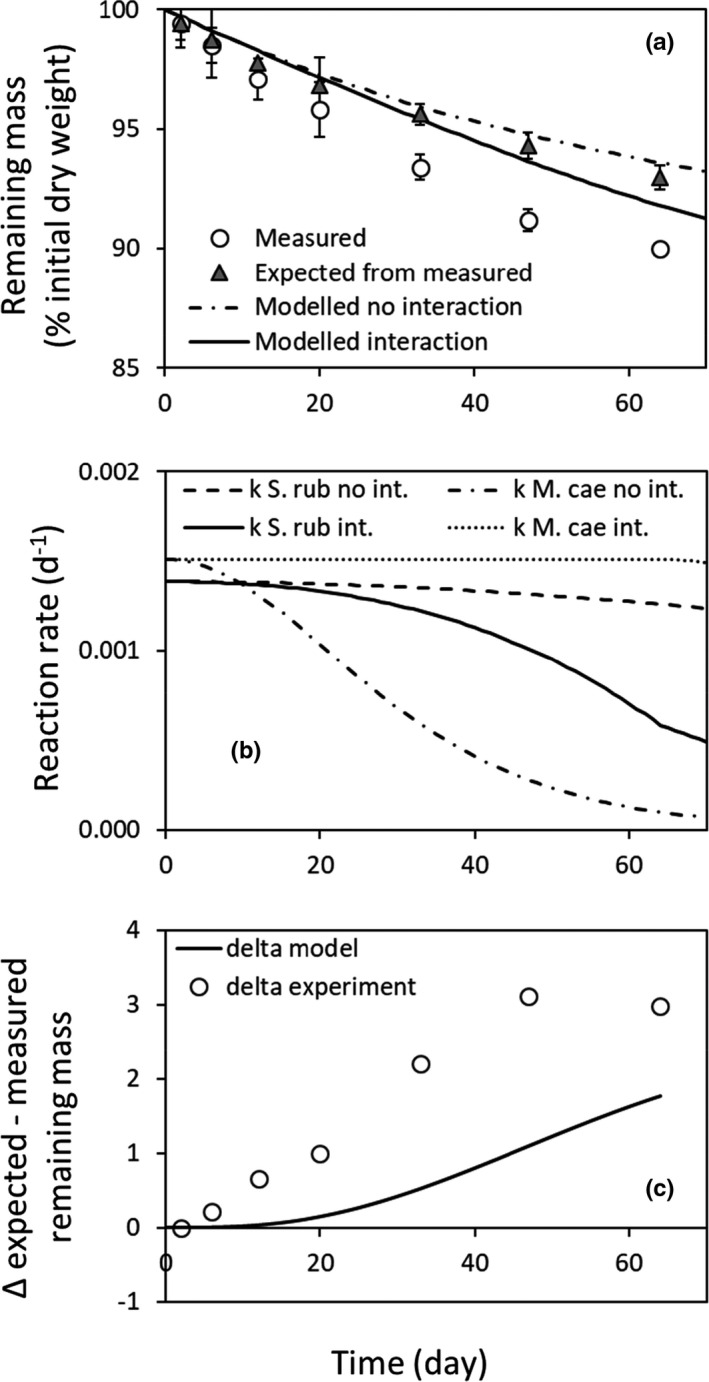
(a) Remaining mass in mixture measured (white dots), expected from litter decomposing alone (additive, gray triangle), modeled with interaction (full line), and modeled without interaction (dotted‐dashed line). (b) Modeled decomposition rate for the *Sphagnum* *rubellum* (*S. rub*) litter with (full gray line) and without interaction (dotted‐dashed gray line), and modeled decomposition rate for the *Molinia caerulea* (*M*. *cae*) litter with (full black line) and without interaction (dotted‐dashed black line). (c) Difference between expected/no interaction and measured/interaction remaining mass (positive value means that a synergistic effect was observed/modeled)

There was a good agreement between the calculated additive remaining mass results from the experiment (remaining mass average between the two litters, the gray triangles, Figure 7a) and the simulation assuming no interactions (dashed line, *R* ^2^ = 0.96, NRMSE = 0.01, Figure 7a). The modeled mass loss assuming interaction (solid line) underestimated the measured mass loss in mixture (white dots, *R* ^2^ = 0.78, NRMSE = 0.04, Figure 7a). As in the numerical experiment, the decrease of reaction rate in the *S*. *rubellum* litter may be overcompensated by the increase of reaction rate in the *M*. *caerulea* litter in the "interaction" situation compared to the "no interaction" case (Figure 7b). In Figure 7c, the delta represents the nonadditive effect in the laboratory experiment and in the numerical simulation (with water exchange between litter) based on single litter data. The difference between expected and measured mass loss in the experiment was higher than what was predicted with the model assuming water flow from the wettest to the driest litter (in this specific case, from *S*. *rubellum* to *M*. *caerulea*, Figure 7c). However, the model does not give account of the totality of the synergistic effect observed in the laboratory experiment (Figure 7c).

## 4 DISCUSSION

### 4.1 Support for the IMC hypothesis and implications

Water content (of soil and litters) is one of the most important factors, along with temperature, in determining the biological activity during the decomposition of litter and thus affecting the rate at which OM is broken down in ecosystems (Chapin et al., 2002; Couteaux et al., 1995). Therefore, litter physical traits related to water content are used to better understand the mechanisms behind the litter mixture effect (Santonja, Baldy, et al., 2015). Soil biological activity needs water as well as compounds dissolved in water such as C substrates and nutrients. However, oxygen availability, which supports aerobic respiration (the most efficient catabolic pathway), decreases as water content increases. This ambivalent effect of water content on soil biological activity is taken into account in models of soil and litter respiration, with an optimal water content set between total desiccation and saturation (Bunnell et al., 1977; Moyano et al., 2013; Sierra et al., 2015). Following Wardle et al. (2003) and Makkonen et al. (2013), we hypothesized in this study that this ambivalent effect could be at the heart of a mechanism triggering a nonadditive mixture effect on litter decomposition. If litter properties determining moisture content were found to be different between interacting litters, flow of water between litters affects individual water content, which can change the rate at which each litter decomposes. Mixing the two litters could either maintain an optimal water content for the two litters around the peak of activity or increase the departure from it.

The numerical simulations showed that a nonadditive effect of mixing litters on decay rate is expected in specific cases depending on the litter moisture content and how it changes with time. First, when the two litters have the same initial LWC and the same decay rate sensitivity to LWC, a synergistic effect can be triggered by different evaporation rates (Figure 5). Different morphological traits that favor water retention can influence the evaporation rates (planar large leaves morphology versus piled small leaves, small versus high ratio of leave perimeter to area). The gradient of LWC between the two litters can generate a flow of water from the wettest litter to the driest one. This maintains both litters in a domain of LWC that sustains a higher decomposition rate than when no water is allowed to flow. This was observed in both models that use a different relationship between biological activity and water content (Figure 5). Second, differences in the dependence of decomposition rate on LWC can also produce a nonadditive effect. A synergistic effect was observed when the litter with the fastest decay rate evaporated at a higher rate than the litter with the lower decomposition rate (Figure 6a,b). When a wet and a slowly decaying litter was allowed to evaporate faster than the other litter (dry and fast decaying litter), an antagonistic effect was observed (Figure 6e,f). This is because the water from the DRY‐LIT in interaction is more rapidly depleted compared the litter with no interaction, as the initially wet litter (WET‐LIT) is rapidly drier than the DRY‐LIT. The increase of WET‐LIT decay rate thanks to the transfer of water is much lower than the decrease of decay rate of DRY‐LIT caused by decreasing water content. Makkonen et al. (2013) assess the water holding capacity not only on single leaves but also on packs of litter, which represents well the micrometeorological conditions in the field. However, no information was given on the rate at which water left these single leaves and packs of litter through evaporation. Future works should be devoted to study the behavior of water in litter packs in single‐species situation and mixture to further understand the important role of water content in litter on OM decomposition.

The calibration showed that, of the two litters studied experimentally, *M*. *caerulea* litter evaporated (coefficient *e*) and potentially decayed faster (*k*_max_) than *S*. *rubellum* litter (Table 2). Furthermore, measurement showed that the gramineae litter had a lower water holding capacity than the bryophyte litter. With such characteristics, theory predicts that a nonadditive synergistic effect on decomposition is possible in the mixture of these two litters. Indeed, the empirical observation (Gogo et al., 2016) confirmed the theoretical prediction as mixing *M*. *caerulea* and *S*. *rubellum* produced a nonadditive synergistic effect on remaining mass (Figure 7). Thus, it is probable that water flows from the *S*. *rubellum* litter to the *M*. *caerulea* litter. This would induce a decrease in the decomposition rate of *S*. *rubellum* litter that was, in proportion to the "no interaction" decomposition rate, lower than the increase of decomposition in the *M*. *caerulea* litter. The results of the simulation suggest that the decrease of the decomposition rate in *S*. *rubellum* was overcompensated by the increase of the decomposition rate of *M*. *caerulea* (Figure 7b). The water flow from *S*. *rubellum* litter to *M*. *caerulea* could allow the microbial communities present in both litters to remain in a domain of litter water content where their activities, and thus the decay rate, remain relatively high for both litters. This result tends to confirm our hypothesis that the shape of the sensitivity of the decay rate to litter water content (inhibitory at both ends) could be at the origin of nonadditive effect on decomposition.

The fact that the water evaporation rate from litter is determinant in explaining the mixture effect has important implications in the present context of climate change. Temperature is increasing leading to more evaporation. Simultaneously, the water regime becomes more and more erratic with an increase in drought periods and intense rainfall episodes, leading to modifications of the soil moisture regime. Such changes can affect the litter mixture effect. Previous studies have indeed shown that a synergistic effect on decomposition could be modulated and even be switched to an antagonistic effect by periods of drought in a Mediterranean forest (Santonja, Fernandez, et al., 2015) and in a temperate grassland (Schuster et al., 2016). Conversely, regular (Iqbal et al., 2015) or increased (Schuster, 2016) rainfall could limit the desiccation period and promote decomposition, which could trigger synergistic effects so far unknown. Finally, as vegetation diversity changes with current disturbances, litter interactions are changing as well as micro‐environmental conditions with effects on litter decomposition (Joly et al., 2017). Consequently, in the context of direct (rainfall intensity, drought period) and indirect (diversity change) effects of climate change on litter decomposition, more attention should be paid to the effect of litter drying/wetting cycles on litter mixture decomposition, coupled to the physical properties affecting litter water content (water holding capacity, evaporation rate at different temperature) of individual leaves and litter in packs. The theoretical results of the present study call for future in situ studies that test whether or not the role of physical properties determining the litter moisture content has any relevance in the field.

### 4.2 Complementary mechanisms of nonadditive effect of mixture on litter decomposition

The validation process showed that the modeled remaining mass from the "no interaction" case fitted well the observed nonadditive expected data from the litter decomposing alone. This suggests that the calibration was good enough to predict experimental data. However, the fit was not as strong for the modeled mixture with interaction and the measured mixture remaining mass (Figure 7a). The difference between expected and measured remaining mass was higher in the laboratory experiment than in the numerical simulation (Figure 7c). This shows that the IMC does not explain the whole synergistic effect observed in these litters. Other factors have to be considered.

When two litters mix, nutrients can flow from one litter to another. For example, in a *Quercus serrata* and *Pinus densiora* mixture, a synergistic effect was triggered by a transfer of nutrients from the nutrient‐rich *Quercus serrata* litter (0.62% of nitrogen—N) to the nutrient‐poor Pinus densiflora litter (0.32% N, Salamanca et al., 1998). However, such a mechanism could not be generalized to all litter types. By using a large panel of litters, Hoorens et al. (2003) showed that two litters with different nutrient contents would not necessarily trigger a nonadditive effect. In a mixture of two decomposing leaves, N flows in both direction (Schimel & Hättenschwiler, 2007). In two mixtures out of the three tested in this last study, N flows from high‐N litter to low‐N litter. In the third case, the high‐N litter, richer in nonstructural carbohydrates than the other high‐N litters, exhibited a high respiration rate and relatively low transfer of N to low‐N litter. Schimel and Hättenschwiler (2007) suggested that as C is not limiting, N will be used by the microbial biomass before being released. Such a "source control" rather than a "gradient control" on N transfer could explain the lack of correlation between N content difference and observed litter mixture effect (Hoorens et al., 2003). The gradient of water between two litters may also be involved in the invalidation of the "gradient control" hypothesis on N transfer. If in a mixture of two litters the driest litter is also the N richer, N transfer from the N‐rich to the N‐poor litter may be less likely to occur as the water flows in the opposite direction. In the present study, *S*. *rubellum* (the wettest litter) contains more N than *M*. *caerulea* (the driest litter, Gogo et al., 2011). Water content and N gradients are in the same direction. Thus, a nutrient effect could occur in this mixture. It would be interesting to carry out further studies involving litter mixtures with different litter N and water content gradients (e.g., mixture of litter with gradients in the same and opposite direction) in order to show how different simultaneous water and N content gradients could combine to affect the nonadditive mixture effect. Furthermore, other nutrient such as P could also be tested, and the Schimel and Hättenschwiler (2007) results also pointed out the need to pay attention to C substrate availability.

To cover their C substrate needs, microbial biomass invests in exo‐enzymes that solubilize solid OM (Schimel & Weintraub, 2003). This soluble C substrate from one litter can feed the microbial biomass present in the other one and trigger a priming effect on this second litter depending on the microbial communities. In the specific case of *S*. *rubellum* and *M*. *caerulea* interactions, such a mechanism could occur. At the beginning of the experiment, *S*. *rubellum* litter may contain a higher amount of soluble monosaccharides than *M*. *caerulea* litter (Gogo et al., 2014). The water flowing from the *S*. *rubellum* litter to the *M*. *caerulea* litter may have contained a significant amount of monosaccharides that may have triggered a priming effect on the microbial biomass dwelling in the *M*. *caerulea* litter. Such an effect, as suggested by Hoorens et al. (2010), could occur in the early stage of decomposition.

Another aspect of C substrate quality deserves more attention in further studies: the litter chemical diversity differences between interacting litters. A more diverse chemical composition leads to an increased probability of compounds and interaction of compounds with a nonadditive effect on biological processes (Chen et al., 2017; Meier & Bowman, 2008). As chemical diversity increases, probability of inhibiting compounds being present also increases (Fierer et al., 2001; Schimel et al., 1998), leading to an antagonistic effect. Conversely, one litter can provide resources (e.g., N and phosphorus—P) that complement those of the other litter leading to the alleviation of a limiting factor. In this situation, increasing chemical diversity leads to a synergistic effect on decomposition (Vos et al., 2013). *Sphagnum* *rubellum* and *M*. *caerulea* litters differ in terms of hydrogen and oxygen content (Gogo et al., 2011) and lipid content (more specifically in terms of some pentacyclic triterpenyl ketones, Zocatelli et al., 2014). Whether these compounds can affect microbial activities remains to be tested.

The chemical diversity of a litter and thus the quality of the C substrate changes with time (Berg & McClaugherty, 2008), which can lead to an antagonistic effect (Butenschoen et al., 2014). Factors controlling decomposition shift with time (García‐Palacios et al., 2016). Depending on the litter types and decomposing environment, the pattern of change of the litter chemistry in the course of decomposition could differ. Three hypothetical controls explaining litter chemical diversity and change were proposed by Wickings et al. (2012): H1—the chemical convergence between two chemically distinct litters due to the fact that common metabolic pathways of distinct microbial communities may lead to a common chemistry, H2—the consistent chemical distinction between two litters due to the "heritage" of the initial litter quality, and H3—the chemistry of a litter could diverge with time due to the occurrence of different microbial communities (Wickings et al., 2012). In the context of litter mixture effect, the control hypothesized in H3 (the decomposer control hypothesis) is particularly relevant as microbial communities can be different between litters and could be transferred from one litter to another. Mixtures of litter exhibiting a synergistic effect on decomposition are good candidates to test such hypotheses. Moyano et al. (2013) have shown that the sensitivity to water content of the microbial activity changes with the availability of substrate. Thus, the C substrate factor is particularly relevant to take into account in future modeling efforts.

## 5 CONCLUSION

Our simulations and observations support the IMC theory that differences in litter properties that are important in determining variation of water content, and especially the evaporation rate, are important factors in accounting for the nonadditive litter mixture effect on decomposition. It is proposed that water flows from the wettest to the driest litter, changing the decomposition rate of the overall mixture. The flow of water from one litter to another can maintain favorable conditions of one litter without reducing in significant extent the decomposition of the other (the litter that provide water). This is done without modification of the whole water content compared to a situation where water does not flow. Our model can be used as a tool to quantify the contribution of litter moisture content in mixture effect. This may be valuable for the understanding of ecosystems functioning in a changing environment. For the specific case studied (mixture of *M*. *caerulea* and *S*. *rubellum*), other factors should be taken into account. Future models and experiments to explore the mechanisms behind the litter mixture effect on decomposition should be focused on (a) testing in the field the theoretical mechanism studied in the present study, (b) the interaction between litter properties related to water content (maximum water content, water holding capacity, evaporation rate) and the chemical (inhibiting compounds, C substrate, mineral nutrients) and microbial diversity, and (c) the effect of in situ drying/wetting cycle frequency and intensity on decomposition.

## CONFLICT OF INTEREST

The authors have no conflict of interest.

## AUTHOR CONTRIBUTIONS

**Sébastien Gogo:** Conceptualization (lead); Data curation (lead); Formal analysis (lead); Funding acquisition (supporting); Methodology (lead); Writing‐original draft (equal). **Fabien Leroy:** Formal analysis (supporting); Methodology (supporting); Writing‐original draft (supporting). **Renata** **Zocatelli:** Formal analysis (supporting); Writing‐original draft (supporting). **Adrien** **Jacotot:** Formal analysis (supporting); Writing‐original draft (supporting). **Fatima** **Laggoun‐Défarge:** Formal analysis (supporting); Funding acquisition (lead); Writing‐original draft (supporting).

## Supporting information

Figure S1‐S2

## Data Availability

The data are available in ZENODO (https://zenodo.org/record/4789262) with the following https://doi.org/10.5281/zenodo.4789262.
