# Supplementary material for: Determinism of nonadditive litter mixture effect on decomposition: Role of the moisture content of litters
Source: Ecol Evol. 2021 Jun 21;11(14):9530–42. doi: 10.1002/ece3.7771 (PMC8293766; doi:10.1002/ece3.7771)
Supplement: Supplementary file 1 — Figure S1‐S2 [file ECE3-11-9530-s001.docx]

**Supplementary material captions**

S1. Observed (white dots) and calibrated (grey lines) litter water content for *Sphagnum rubellum* (A) and *Molinia caerulea* (C) litter. Relationship between Fitted versus observed water content for *Sphagnum rubellum* (B) and *Molinia caerulea* (D) litter, with R^2^_adj_ = adjusted R square, NRMSE = normalised root mean square error.

S2. Observed (white dots) and calibrated (grey lines) remaining mass for *Sphagnum rubellum* (A) and *Molinia caerulea* (C) litter. Relationship between Fitted versus observed remaining mass for *Sphagnum rubellum* (B) and *Molinia caerulea* (D) litter, with R^2^ adj = adjusted R square, NRMSE = normalised root mean square error.

**Supplementary materials**


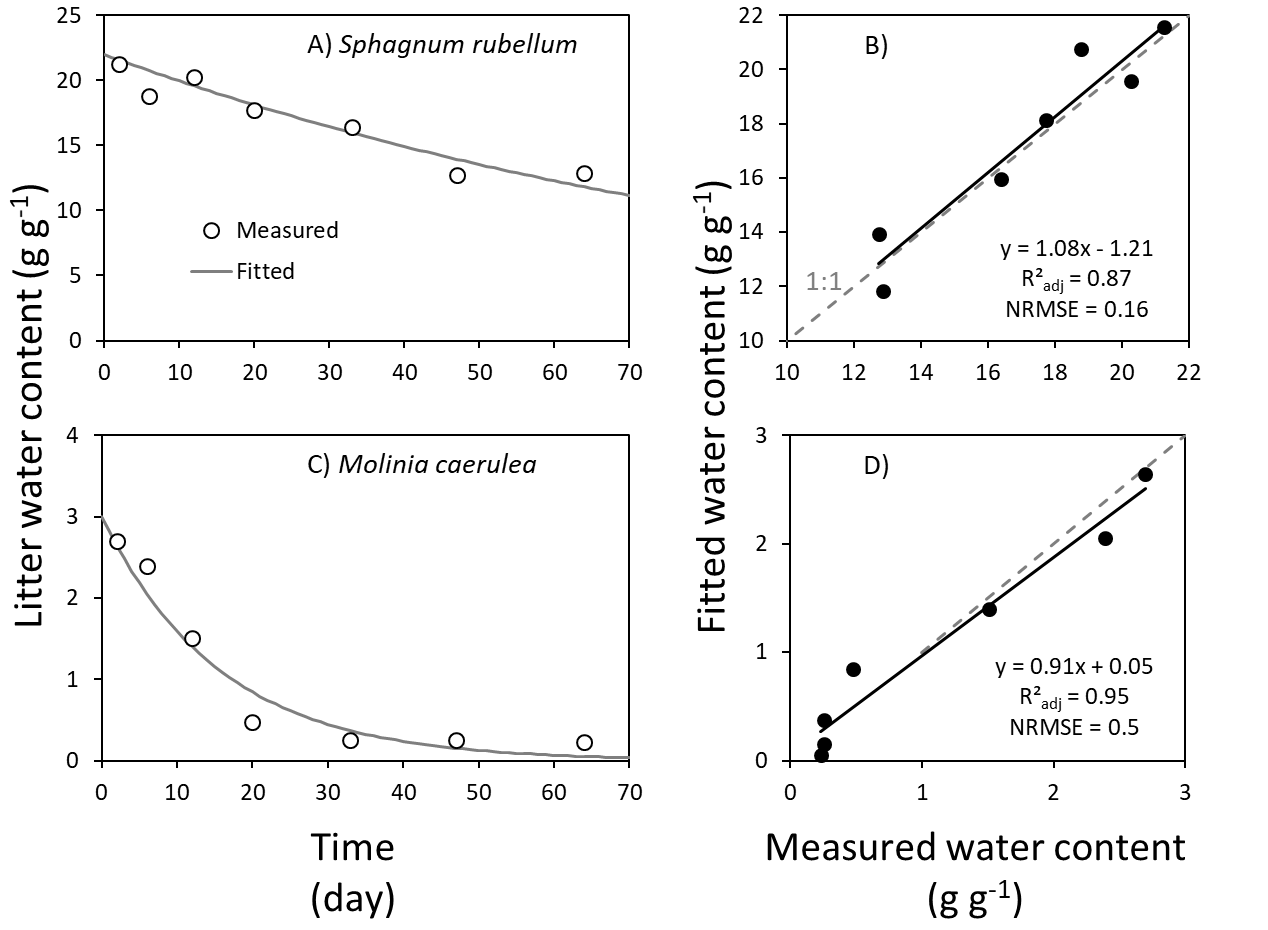


S1.


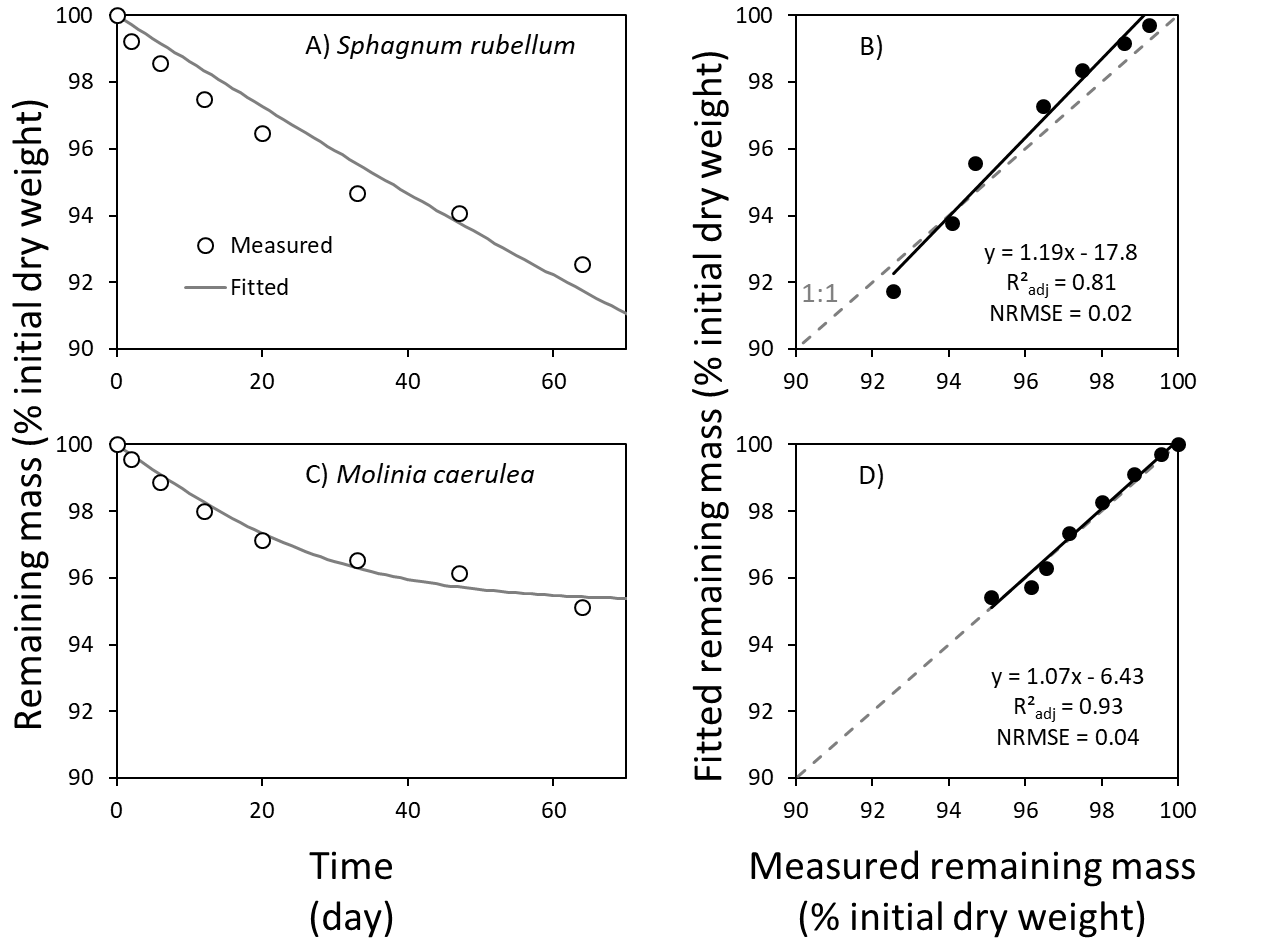


S2.
